# Sustained low micromolar hydrogen peroxide exposure induces sequential red blood cell dysfunction

**DOI:** 10.3389/fphys.2026.1860905

**Published:** 2026-07-10

**Authors:** Jin Hyen Baek, Matthew C. Williams, Sirsendu Jana, Xiaoyuan Zhang, Felice D’Agnillo

**Affiliations:** Laboratory of Biochemistry and Vascular Biology, Office of Blood Research and Review, Center for Biologics Evaluation and Research (CBER), U.S. Food and Drug Administration (FDA), Silver Spring, MD, United States

**Keywords:** biomarkers, erythrocyte deformability, glutathione depletion, hemoglobin oxidation, membrane integrity, oxidative stress, phosphatidylserine, vesiculation

## Abstract

**Introduction:**

Red blood cells (RBCs) are continuously exposed to oxidative stress throughout their lifespan and during *ex vivo* storage. Most experimental oxidative stress models rely on supraphysiological bolus oxidant addition, yet this approach does not replicate the sustained, low-level oxidant exposure encountered physiologically. Understanding the temporal sequence of RBC oxidative injury under physiologically relevant conditions is essential for identifying early biomarkers of dysfunction and developing targeted interventions.

**Methods:**

We employed a glucose oxidase (GX)-based system to generate sustained hydrogen peroxide (H_2_O_2_) at low micromolar concentrations (0.8–8 µM) and examined the temporal progression of oxidative damage in human RBCs over 24 hours. Healthy donor RBCs exposed to GX (1–10 mU/mL) were evaluated for oxidative burden, antioxidant status, hemoglobin oxidation, relative hemoglobin release, membrane integrity/viability by calcein fluorescence, deformability by ektacytometry, phosphatidylserine (PS) externalization by Annexin V binding, and vesiculation by microscopy.

**Results:**

Sustained H_2_O_2_ exposure induced rapid glutathione depletion (within 6 hours) followed by progressive methemoglobin formation. Single-cell analyses demonstrated a strong inverse relationship between intracellular oxidative autofluorescence and cellular dysfunction. GX induced concentration-dependent impairment of RBC deformability, with 10 mU/mL causing significant membrane rigidity and hemoglobin release indicative of membrane lysis by 24 hours. Time-dependent vesiculation and release of CD235a- and Band 3-labeled microvesicles occurred with vesiculating RBCs exhibiting higher oxidative burden than non-vesiculating cells. Notably, PS externalization was absent on both vesiculating RBCs and their shed microvesicles.

**Discussion:**

These findings define a temporal hierarchy of oxidative injury under physiologically relevant conditions and demonstrate that vesiculation and PS externalization are mechanistically uncoupled under sustained oxidative stress. This model provides a framework for identifying early biomarkers of RBC dysfunction that may guide the development of targeted interventions to optimize blood storage and mitigate oxidative injury.

## Graphical Abstract

**Figure:**
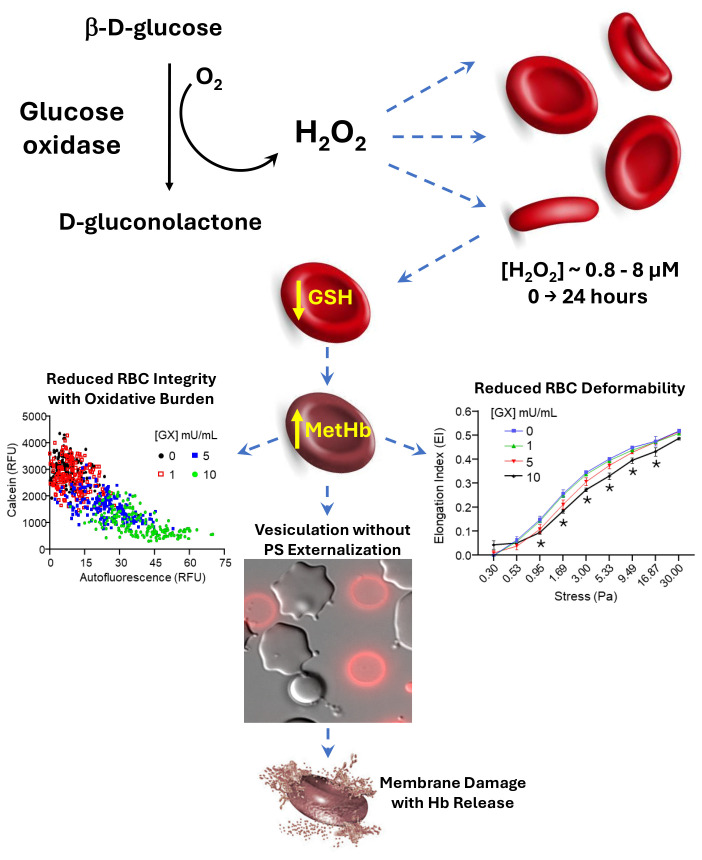

## Introduction

Red blood cells (RBCs) are highly specialized cells responsible for efficient oxygen transport and carbon dioxide removal, and their structural and biochemical integrity is essential for effective microvascular perfusion. Because RBCs continuously cycle between oxygenated and deoxygenated states driven by high concentrations of iron (Fe^2+^)-rich hemoglobin, they are intrinsically susceptible to oxidative stress (Kuhn et al., 2017; Alayash, 2022; Spinelli et al., 2025). Reactive oxygen species (ROS), including superoxide and hydrogen peroxide (H_2_O_2_), are generated endogenously through hemoglobin autoxidation and cellular metabolism, and are also encountered from extracellular inflammatory and vascular sources (Spinelli et al., 2025; Alayash, 2022; Daraghmeh and Karaman, 2024). Although RBCs possess robust antioxidant systems—most notably glutathione (GSH), catalase, glutathione peroxidase, and peroxiredoxin-2—these defenses must counterbalance persistent oxidant exposure in the absence of *de novo* protein synthesis or organellar repair mechanisms (Kuhn et al., 2017; Forman et al., 2016; Daraghmeh and Karaman, 2024; Spinelli et al., 2025).

Physiological steady-state concentrations of H_2_O_2_ in human blood range from approximately 0.8 to 6 μM, subjecting RBCs to a chronic, low-level oxidant flux (Forman et al., 2016; Gaikwad et al., 2021). During pathological states (e.g., inflammation, sepsis, hemoglobinopathies) or refrigerated blood storage, this balance shifts toward cumulative oxidative injury—which can lead to membrane remodeling, metabolic dysregulation, vesiculation, and reduced post-transfusion recovery (Yoshida et al., 2019; Kassa et al., 2024; Baek et al., 2012; Hess, 2010; Johnson et al., 1994). Extensive *in vitro* studies using supraphysiologic bolus additions of oxidants, with or without catalase inactivation, have identified multiple consequences of oxidative injury. These include lipid and hemoglobin oxidation, ATP depletion, calcium influx, Band 3 clustering, PS externalization, loss of deformability, eryptosis, and ultimate membrane lysis (Benincasa et al., 2025; Sinha et al., 2015; Russo et al., 2024; Snyder et al., 1985; Hale et al., 2011; Larkin et al., 2024; Nagababu et al., 2003; Sudnitsyna et al., 2020; Orrico et al., 2023; Rinalducci et al., 2012; Brun et al., 2021). However, these acute, high-concentration models fail to replicate the sustained, low-level oxidant exposure encountered *in vivo* or during storage. Consequently, such acute challenges likely obscure early adaptive responses, leaving the precise mechanistic sequence linking initial antioxidant depletion to cytoskeletal destabilization, vesiculation, and late-stage membrane failure incompletely defined.

RBC vesiculation is an important but incompletely understood consequence of oxidative stress (Leal et al., 2018; Mohanty et al., 2014). During their 120-day lifespan, RBCs progressively shed 100–200 nm microvesicles, contributing to removal of damaged components and preservation of surface area-to-volume ratio. Oxidative modification weakens spectrin-actin cytoskeletal interactions and promotes membrane budding, often with PS externalization and altered calcium homeostasis (Leal et al., 2018; Nguyen et al., 2016). However, the precise temporal succession of these events and the mechanistic links between oxidative burden and vesicle formation remain unresolved.

A critical knowledge gap therefore exists in defining the hierarchical progression of RBC injury under physiologically relevant oxidant flux. Specifically, it remains unclear which alterations represent the earliest and most sensitive indicators of dysfunction, how antioxidant depletion quantitatively relates to hemoglobin oxidation and biomechanical impairment, and the temporal interplay among the loss of membrane asymmetry, vesiculation, and complete membrane failure.

To address these gaps, we examined a glucose oxidase (GX)-based *in vitro* system that generates sustained, low micromolar concentrations of H_2_O_2_, thereby mimicking physiologic oxidant exposure more closely than bolus models. Using this platform, we performed a comprehensive temporal analysis of human RBC oxidative injury, integrating measurements of extracellular H_2_O_2_, intracellular GSH depletion, methemoglobin formation, deformability by ektacytometry, membrane integrity, vesiculation, PS externalization, and hemoglobin release. By defining the temporal hierarchy and interrelationship of these events, this study identifies early biomarkers of RBC dysfunction and provides mechanistic insight relevant to the blood storage lesion and oxidative stress–associated hematologic disorders.

## Materials and methods

### RBC culture and experimental treatments

Human whole blood was obtained from healthy volunteers through the American Red Cross (Rockville, MD, USA) under protocols reviewed and approved by the American Red Cross Institutional Review Board (IRB). Blood was collected in storage bags containing citrate phosphate dextrose (CPD) anticoagulant, and written informed consent was obtained from all donors. RBCs were isolated from whole blood units (≤ 4 days old) by centrifugation at 300 × g for 10 minutes at room temperature using an Eppendorf Model 5430R centrifuge (Eppendorf AG, Hamburg, Germany). Plasma and buffy coat were removed by aspiration, and RBCs were resuspended with Dulbecco’s Phosphate-Buffered Saline (DPBS; Cat. No. 14287080, Thermo Fisher Scientific, Waltham, MA, USA) supplemented with 5.5 mM D-glucose. RBC concentrations were determined using a Countess II automated cell counter (Thermo Fisher Scientific), adjusted to a final concentration of 1.65 – 1.75 × 10^6^ cells/mL, and plated at 1 mL per well in 24-well cell-repellent plates (Greiner Bio-One, Monroe, NC, USA) or 4-well poly-L-lysine-coated polymer coverslips (Ibidi GmbH, Fitchburg, WI, USA). Following a 20-minute equilibration period at room temperature, RBCs were treated with varying concentrations of glucose oxidase (GX; Cat. No. G7141, Type X-S from *Aspergillus niger*, 100,000–250,000 units/g solid; MilliporeSigma, St. Louis, MO, USA). GX catalyzes the oxidation of β-D-glucose to D-glucono-1,5-lactone while simultaneously reducing molecular oxygen to H_2_O_2_. Treated cultures were incubated at room temperature for up to 24 hours, with samples collected at designated time points for subsequent analysis.

### Hydrogen peroxide determination

Extracellular H_2_O_2_ concentrations in RBC cultures were quantified using the Amplex Red Glucose/Glucose Oxidase Assay Kit (Cat. No. A22189, Thermo Fisher Scientific, Waltham, MA, USA). RBCs were cultured in 24-well plates under indicated conditions. Culture supernatants (50 µL) were mixed with an equal volume of reaction mixture (50 µM Amplex^®^ Red reagent and 0.1 U/mL HRP in 50 mM sodium phosphate buffer, pH 7.4) in a 96-well plate. Absorbance at 560 nm was monitored for 20 minutes at room temperature using a BioTek Synergy 4 microplate reader (BioTek Instruments, Winooski, VT, USA). The absorbance value recorded at the 10-minute time point was used for quantification purposes with DPBS alone controls included for background subtraction. H_2_O_2_ concentrations were calculated by comparison to a standard curve generated using known concentrations of H_2_O_2_ prepared and measured under identical assay conditions.

### Intracellular glutathione determination

Intracellular glutathione (GSH) concentrations were quantified using the GSH-Glo™ Glutathione Assay (Promega Corporation, Madison, WI, USA) according to the manufacturer’s protocol with minor modifications. RBCs cultured in 24-well plates were gently resuspended by pipetting, and 50 µL aliquots were transferred to a 96-well white opaque microplate (Costar Cat. No. 3912, Corning Inc., Corning, NY, USA). The 2× GSH-Glo™ reaction buffer was prepared by combining 1 mL of reconstituted GSH-Glo™ reaction buffer with 20 µL of glutathione S-transferase and 20 µL of luciferin-NT substrate. An equal volume (50 µL) of the 2× reaction buffer was added to the cell suspension (final volume of 100 µL per well). The plate was incubated for 30 minutes at room temperature in the dark to allow for GSH-dependent conversion of the luciferin derivative. Following this incubation period, 100 µL of reconstituted Luciferin Detection Reagent was added to each well, and the plate was incubated for an additional 15 minutes at room temperature in the dark to stabilize the luminescent signal. Luminescence was measured using a BioTek Synergy 4 microplate reader. GSH levels were determined using a standard curve of known GSH concentrations. All samples were analyzed in duplicate or triplicate, and background luminescence from cell-free wells was subtracted from all measurements.

### Methemoglobin and hemoglobin release measurement

Intracellular methemoglobin content and relative hemoglobin release were assessed in RBCs cultured in 24-well plates. For methemoglobin determination, culture supernatants were aspirated, and RBCs were lysed in distilled water for 10 minutes at room temperature. Lysates were transferred to 96-well plates for spectrophotometric analysis using a BioTek Synergy 4 plate reader. Total hemoglobin concentration and the percentage of methemoglobin were calculated by multicomponent analysis using absorbance readings at 576, 560, 630, and 700 nm (Butt et al., 2010). For measurement of hemoglobin release, 200 µL of supernatant from each well was transferred to a 96-well plate and absorbance was measured spectrophotometrically at 405 nm. Total hemoglobin absorbance at 405 nm was obtained from corresponding untreated wells following lysis of RBCs with distilled water for 10 minutes. Relative hemoglobin release was calculated using the following equation: Hemoglobin Release (%) = [(ABS_405_ Supernatant - ABS_405_ Blank)/(ABS_405_ Total Hb - ABS_405_ Blank)] × 100.

### Imaging analysis of RBC autofluorescence and calcein fluorescence

Dual-color fluorescence microscopic imaging of RBC autofluorescence and Calcein Deep Red were evaluated as indicators of oxidative damage and cellular viability and/or membrane integrity, respectively (Bratosin et al., 2005; Stoya et al., 2002). Following experimental treatments, unfixed RBCs in poly-L-lysine-coated 4-well chambered coverslips were incubated with 5 µM Calcein Deep Red™ AM ester (Cat. No. C22011, AAT Bioquest, Sunnyvale, CA, USA) for 30 minutes at room temperature in the dark. The acetoxymethyl (AM) ester form of the dye passively diffuses across intact cell membranes and is cleaved by intracellular esterases to generate the fluorescent, membrane-impermeant Calcein Deep Red product, which is retained in cells with intact membranes; thus, this technique assesses cellular viability and/or membrane integrity, as calcein retention requires both functional esterases and intact membranes. Following incubation, unincorporated dye was removed by gently washing the coverslips two times with DPBS, and fresh DPBS was added to the wells for imaging.

Coverslips were examined using an Axio Observer Z1 inverted microscope (Carl Zeiss Microscopy, Thornwood, NY, USA) equipped with an Axiocam 506 monochrome camera and an ApoTome.2 optical sectioning system for enhanced image resolution. Images were acquired using a Plan-Apochromat 63×/1.4 NA oil immersion objective (working distance = 0.19 mm) for high-resolution single-cell analysis for field-of-view imaging. RBC autofluorescence was detected using the FITC filter set (excitation: 450–490 nm; emission: 500–550 nm), while Calcein Deep Red fluorescence was detected using the Cy5 filter set (excitation: 625–655 nm; emission: 665–715 nm). For each experimental condition, multiple fields of view were captured, and exposure times were kept constant across all samples to enable quantitative comparisons. Digital image processing and quantitative fluorescence analysis were performed using ZEN 2 software (version 2.0, Carl Zeiss Microscopy). Individual RBCs were manually segmented, and fluorescence intensity values were measured for both autofluorescence and Calcein Deep Red channels. Ghost cells, identified by their characteristic morphology and complete absence of Calcein Deep Red retention, were excluded from quantitative fluorescence analysis of intact RBCs. For each experimental condition, mean fluorescence intensity values were derived from a minimum of 50–100 individual cell images acquired across multiple independent experiments. Background fluorescence was subtracted from all measurements.

### Imaging analysis of RBC microvesicles

After experimental treatments, RBCs in chambered coverslips were fixed for 10 minutes at room temperature with 0.1% glutaraldehyde and 1% paraformaldehyde in PBS (Electron Microscopy Sciences, Hatfield, PA, USA). Coverslips were washed three times with PBS, permeabilized with 0.1% Triton X-100 in PBS for 12 minutes, and blocked with 1% BSA in PBS for 20 minutes. For immunofluorescence labeling of RBC membrane proteins, cells were incubated with primary antibodies against Band 3 (mouse monoclonal, Cat. No. MA1-20211, Thermo Fisher Scientific, Waltham, MA, USA) and CD235a/glycophorin A (rabbit polyclonal, Cat. No. PA5-141179, Thermo Fisher Scientific) in PBS containing 1% BSA for 1 hour at room temperature. Coverslips were then washed and incubated with Alexa Fluor 488-conjugated donkey anti-mouse IgG (Cat. No. A-21206) and Alexa Fluor 647-conjugated donkey anti-rabbit IgG (Cat. No. A-31571) secondary antibodies (Thermo Fisher Scientific) for 30 minutes in the dark. Coverslips were washed three times with PBS and mounting medium was added for imaging.

Coverslips were examined using an Axio Observer Z1 inverted microscope equipped with an Axiocam 506 monochrome camera and an ApoTome.2 optical sectioning system. Images were acquired using a Plan-Apochromat 63×/1.4 NA oil immersion objective (working distance = 0.19 mm). Multiple fields of view were captured with consistent exposure settings across all samples. Z-stack images were acquired at 0.3–0.5 µm intervals to visualize microvesicles that were typically < 1 µm in diameter and remained adjacent or loosely attached to the parent RBC.

### Phosphatidylserine externalization analysis by flow cytometry and microscopy

Flow cytometry was performed using a spectral Aurora flow cytometer (Cytek Biosciences, Fremont, CA, USA), with a minimum of 50,000 events acquired per sample. Membrane PS externalization was detected using Annexin V-Alexa Fluor 647 conjugate (Cat# A23204, Thermo Fisher Scientific, Waltham, MA, USA). Culture medium was aspirated, and Annexin V-conjugate was added in 0.5 mL HEPES binding buffer (10 mM HEPES, 140 mM NaCl, 2.5 mM CaCl_2_, pH 7.4). After incubation for 15 minutes at 25 °C in the dark, 0.5 mL binding buffer was added. RBCs were transferred to flow cytometry tubes for immediate analysis. Data were acquired using SpectroFlo software v3.0 (Cytek Biosciences) and analyzed using FlowJo software v10.8.1 (BD Life Sciences, Ashland, OR, USA). RBC populations were identified by FSC/SSC characteristics with debris excluded by gating. Annexin V-positive cells were identified by fluorescence intensity in the Alexa Fluor 647 channel, with gates set using unstained controls. Results are expressed as the percentage of Annexin V-positive cells within the gated RBC population. For live-cell imaging of PS externalization, unfixed RBCs were incubated with 15 µg/mL Alexa Fluor 647-conjugated Annexin V in HEPES binding buffer for 20 minutes at room temperature in the dark. Following incubation, cells were gently washed with binding buffer, and fresh binding buffer was added to the wells for immediate imaging by fluorescence microscopy.

### Assessment of RBC deformability by laser diffraction ektacytometry

RBC deformability was quantified using laser diffraction ektacytometry (LORRCA MaxSis, RR Mechatronics, Hoorn, The Netherlands) at multiple fluid shear stress levels. Following experimental treatments, RBCs cultured in 24-well plates were harvested by gentle pipetting and centrifuged at 300 × g for 5 minutes at room temperature. The cell pellet was resuspended in Elon-ISO viscous medium (RR Mechatronics) at an appropriate hematocrit to ensure optimal light scattering properties. Shear stress-elongation index (EI) curves were generated at 37 °C by subjecting the RBC suspension to nine incremental shear stress values ranging from 0.3 to 30 Pa. At each shear stress level, a monochromatic laser beam (wavelength 670 nm) was transmitted through the sheared sample, and the resulting diffraction pattern produced by the deformed RBCs was captured by the instrument’s charge-coupled device (CCD) camera. The EI was calculated from the geometric parameters of the elliptical diffraction pattern according to the equation: EI = (L − W)/(L + W), where L represents the major axis (length) and W represents the minor axis (width) of the diffraction ellipse. Higher EI values at a given shear stress indicated greater cellular elongation and, consequently, enhanced RBC deformability. For each experimental condition, mean EI values were plotted as a function of applied shear stress to generate complete deformability profiles.

### Statistical analysis

Data values are expressed as mean ± SEM unless indicated otherwise. Statistical comparisons were performed by a one-way ANOVA for planned comparisons and *post-hoc* Bonferroni’s test for multiple comparisons between equal size groups or Dunnett’s post-test when group comparisons involved unequal sizes (GraphPad Prism 10 software, La Jolla, CA). The minimum level of statistical significance was p < 0.05.

## Results

### Low micromolar H_2_O_2_ generation by glucose oxidase

**Figure 1:**
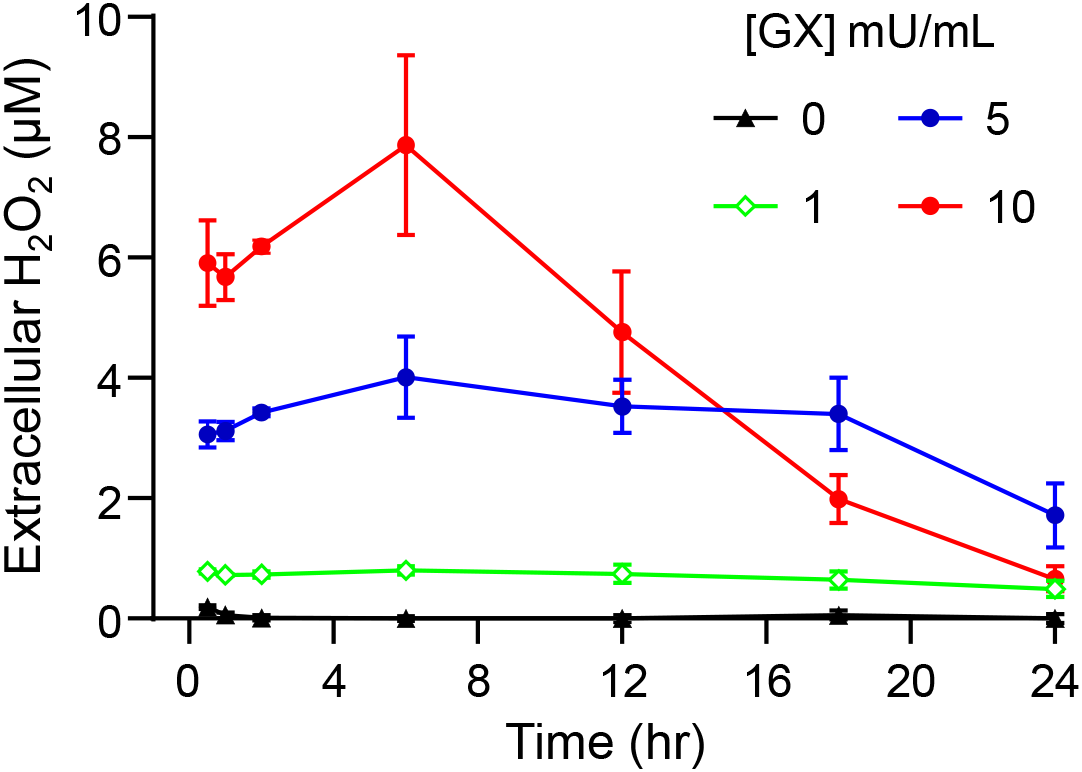
Extracellular H_2_O_2_ generation in RBC cultures. Human RBCs were incubated in Dulbecco’s phosphate-buffered saline (DPBS) alone or in the presence of 1, 5, or 10 mU/mL GX concentrations. Extracellular H_2_O_2_ concentrations were measured at each indicated time point as described in Materials and Methods. The first reading was taken at 30 min. All GX concentrations produced significantly elevated extracellular H_2_O_2_ levels compared to DPBS alone at all time points (p < 0.001). Each value represents the mean ± SEM for 4 independent experiments performed in triplicate (n = 4 individual donors).

To establish a controlled model of sustained oxidative stress, extracellular H_2_O_2_ production was quantified in RBC cultures treated with increasing concentrations of glucose oxidase (GX; 1, 5, and 10 mU/mL). Extracellular H_2_O_2_ levels were measured at multiple time points beginning at 30 minutes post-treatment. All tested GX concentrations resulted in significantly elevated extracellular H_2_O_2_ compared to control cells incubated in DPBS alone (p < 0.001) (**Figure 1**). Peak H_2_O_2_ levels were observed between 4–8 hours, reaching approximately 7–8 µM in the 10 mU/mL group, 3–4 µM in the 5 mU/mL group, and 0.8 µM in the 1 mU/mL group. H_2_O_2_ concentrations in the 1 and 5 mU/mL groups remained relatively stable through 24 hours, whereas concentrations in the 10 mU/mL group declined after 8 hours, decreasing to < 1µM by 24 hours. In control experiments with 10 mU/mL GX added to culture dishes without RBCs, H_2_O_2_ concentrations remained elevated over 24 hours (**Supplementary Figure 1A**), indicating that the decline observed in RBC-containing cultures at this GX concentration was attributable to accelerated consumption of extracellular H_2_O_2_ rather than loss of enzyme activity. Substrate depletion is also unlikely to account for the observed H_2_O_2_ decline, as glucose concentrations in RBC culture supernatants remained around 4.5 mM (from 5.5 mM) over 24 hours with 10 mU/mL GX (**Supplementary Figure 1B**).

### Sustained H_2_O_2_ exposure depletes intracellular glutathione

**Figure 2:**
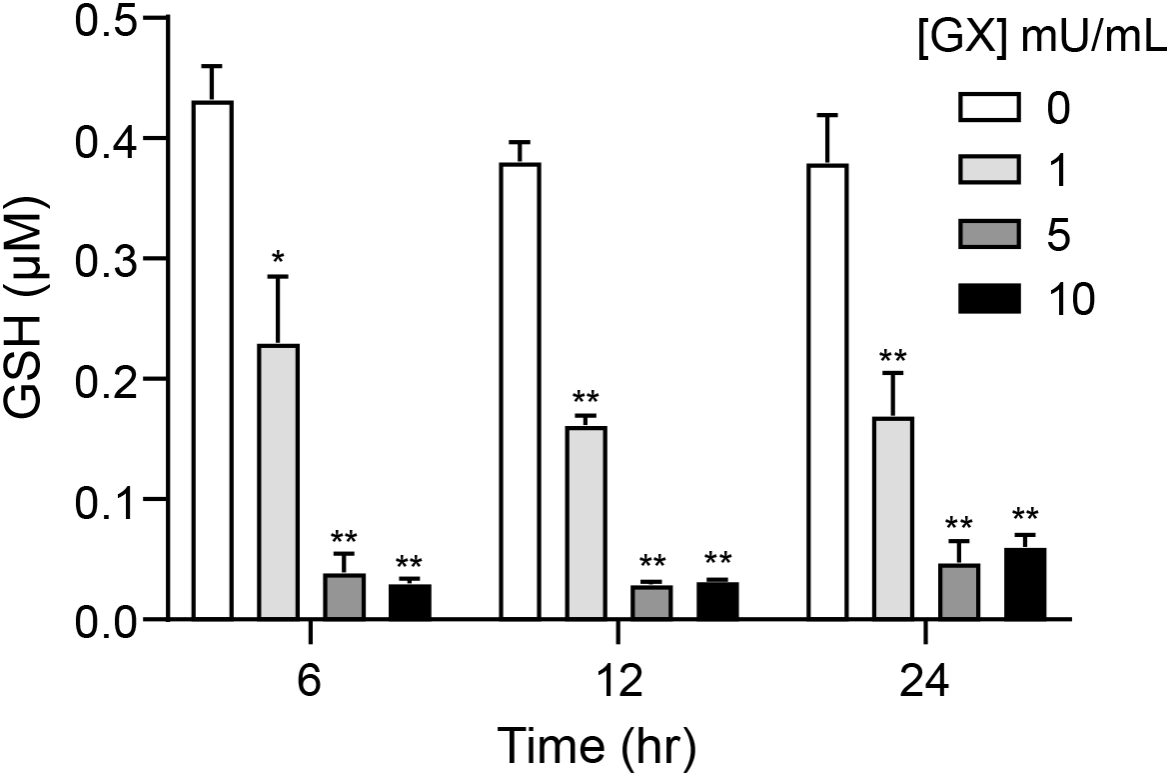
Intracellular GSH levels following GX exposure. RBCs were incubated with DPBS alone or in the presence of 1, 5, or 10 mU/mL GX for 6, 12, and 24 hours. Intracellular GSH levels at each time point were analyzed as described in Materials and Methods. GSH concentrations are derived from a standard curve and reported as the means ± SEM for a minimum of four independent experiments performed in triplicate (n = 4 individual donors). * p < 0.05, ** p < 0.001 vs. DPBS alone.

Intracellular glutathione (GSH) levels were measured at 6, 12, and 24 hours to assess antioxidant depletion (**Figure 2**). In DPBS alone, GSH concentrations remained stable across all time points. In contrast, both 5 and 10 mU/mL GX reduced GSH to <0.1 µM across all time points. The 1 mU/mL group produced significant reductions in GSH levels to approximately 0.2 μM across all time points. All GX-treated groups were significantly lower than controls at indicated time points (p < 0.001). These findings indicate rapid and substantial GSH exhaustion under sustained low-level oxidative stress conditions, establishing a cellular environment vulnerable to subsequent oxidative damage.

### Methemoglobin formation and reduced viability/membrane integrity

**Figure 3:**
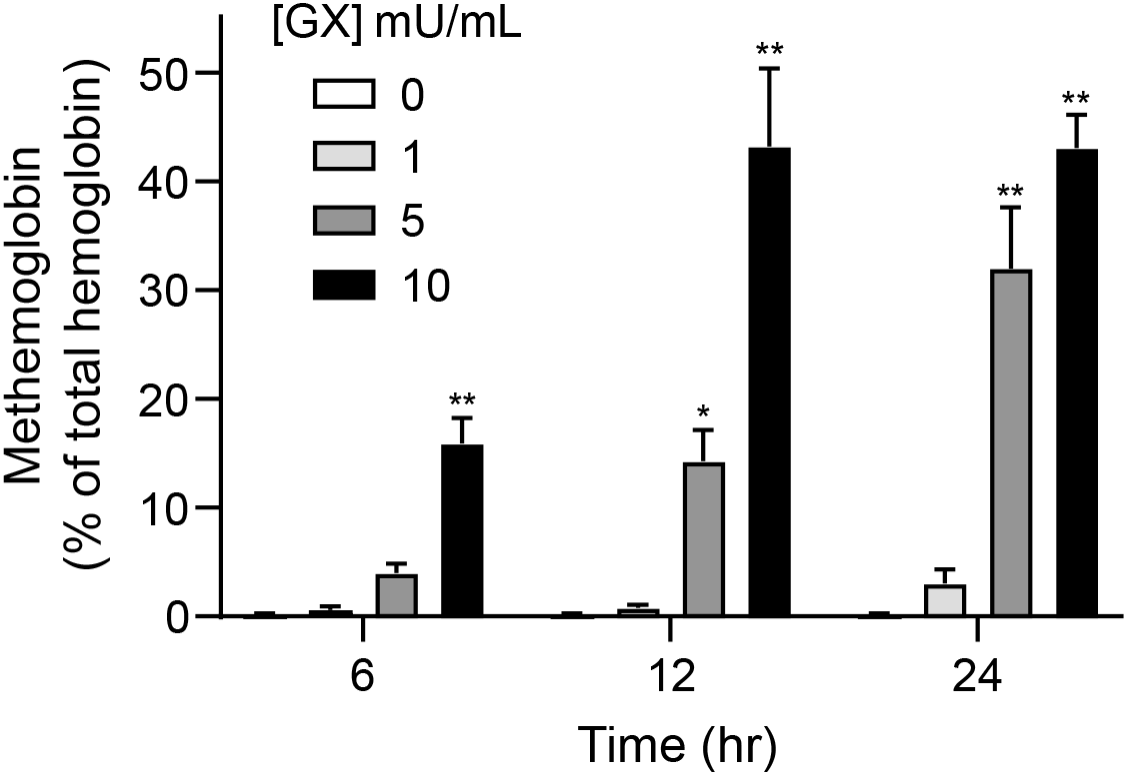
Methemoglobin formation following GX exposure. Human RBCs were incubated with DPBS alone or in the presence of 1, 5, or 10 mU/mL GX. The percentage of intracellular methemoglobin was measured after 6, 12, and 24 hours. Each value represents the mean ± SEM for a minimum of four independent experiments (n = 4–5 individual donors). * p < 0.05, ** p < 0.001 vs. DPBS alone.

The impact of GX-induced oxidative stress on RBC molecular components was evaluated by measuring intracellular methemoglobin formation at 6, 12, and 24 hours. Methemoglobin levels increased in both a concentration- and time-dependent manner (**Figure 3**). At 6 hours, MetHb levels rose modestly in the 5 mU/mL GX group and more substantially in the 10 mU/mL group, while the 1 mU/mL group showed minimal elevation. By 24 hours, 10 mU/mL GX resulted in MetHb levels approaching ~40–45%, compared to ~30-35% with 5 mU/mL and 2-3% with 1 mU/mL, whereas DPBS controls remained <2%. These results demonstrate that sustained H_2_O_2_ exposure induces progressive hemoglobin oxidation in a time- and concentration-dependent manner.

**Figure 4:**
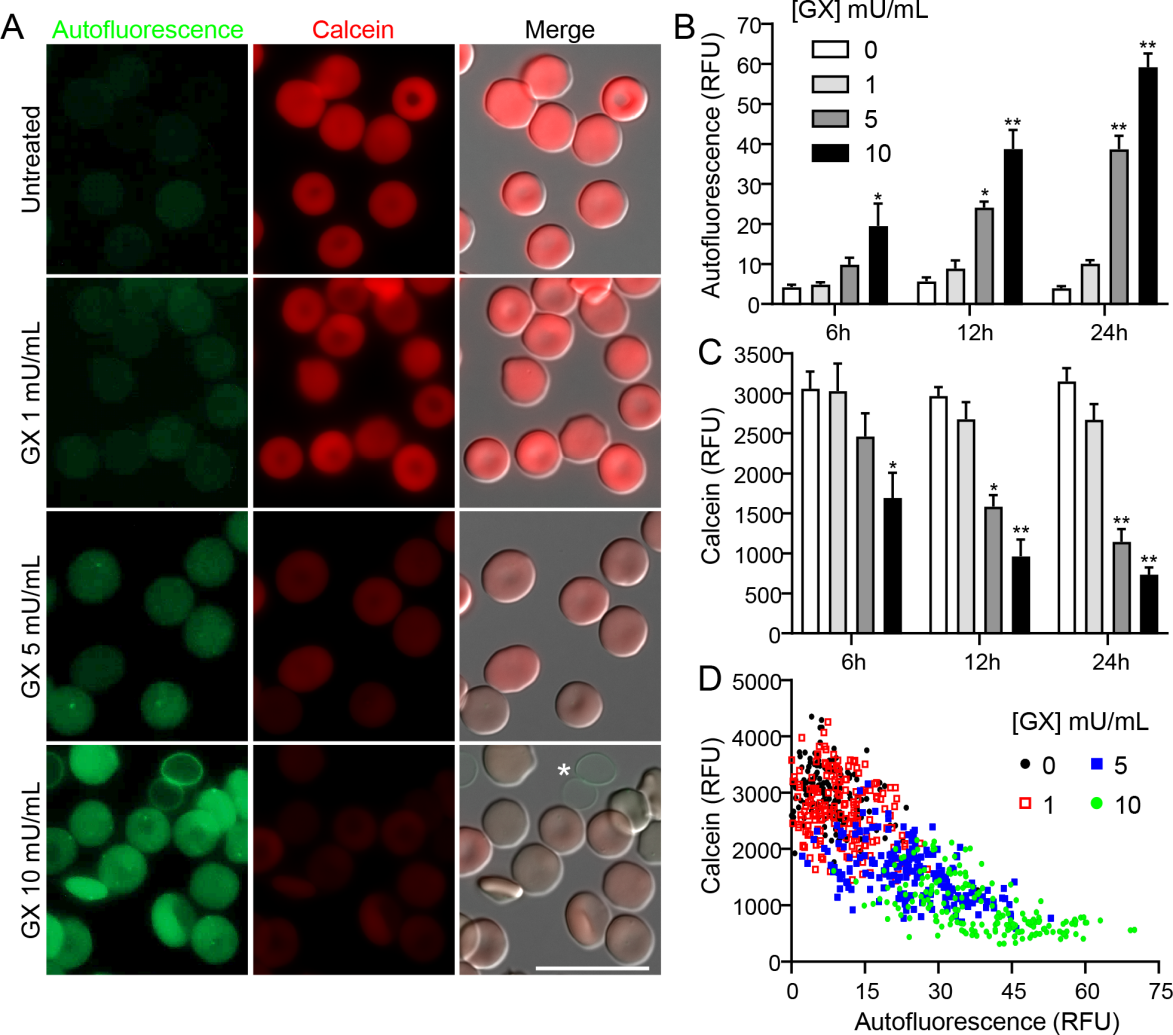
Oxidative damage and viability and/or membrane integrity following GX exposure. Human RBCs cultured in chamber slides were treated with increasing GX concentrations. Unfixed RBCs at the indicated time intervals were imaged simultaneously for RBC autofluorescence as a marker of oxidative damage and calcein fluorescence as a marker of viability and/or membrane integrity. **(A)** Representative fluorescence images of RBC autofluorescence (FITC settings) and intracellular calcein (Cy5 settings) in untreated and GX-treated RBCs after 24 hours. Asterisk denotes two ghost cells with no calcein signal. Scale bar = 20 µm. Quantitative analysis of relative fluorescence intensity (RFU) for **(B)** autofluorescence and **(C)** calcein were reported as the mean RFUs ± SEM for a minimum of four independent experiments performed in duplicate. **(D)** Correlation between RBC autofluorescence and intracellular calcein signal intensity of each individual RBC analyzed at each GX concentration for the 12-hour interval. Each data point represents an individual RBC. A strong inverse correlation was observed (Spearman’s ρ = -0.83, p < 0.001, n = 732 cells). *p < 0.05, **p < 0.001 vs. DPBS alone.

To examine the relationship between oxidative damage and cellular viability and/or membrane integrity, RBC autofluorescence and intracellular calcein were evaluated by dual-color fluorescence microscopy. Marked oxidative autofluorescence accumulation and reduced intracellular calcein signal were observed in GX-treated RBCs relative to untreated controls (**Figure 4A**). Quantitative image analysis revealed concentration- and time-dependent increases in autofluorescence, reaching approximately 60–70 RFU at 24 hours in the 10 mU/mL group compared with <10 RFU in DPBS alone (**Figure 4B**). The 5 mU/mL group exhibited intermediate increases (~30–40 RFU), while 1 mU/mL induced modest elevations. Conversely, calcein fluorescence declined progressively with GX exposure (**Figure 4C**). At 24 hours, intracellular calcein fluorescence decreased to approximately 500–1000 RFU in the 10 mU/mL group compared to ~3000 RFU in DPBS alone. The 5 mU/mL group exhibited progressive reductions from 2500 to 1000 RFU at 24 hours, while 1 mU/mL induced modest decreases. Ghost cells without detectable calcein signal were excluded from quantitative analysis. Single-cell correlation analysis at 12 hours demonstrated a strong inverse relationship between RBC autofluorescence and calcein signal intensity (Spearman’s ρ = -0.83, p < 0.001, n = 732 cells), with higher oxidative signal corresponding to reduced viability and/or membrane integrity (**Figure 4D**). Together, these findings demonstrate that sustained low micromolar H_2_O_2_ exposure produces progressive oxidative damage to hemoglobin and other cellular components, that corresponds with loss of viability (reduced intracellular esterase activity) and/or loss of membrane integrity.

### Sustained H_2_O_2_ triggers localized microvesicle formation

**Figure 5:**
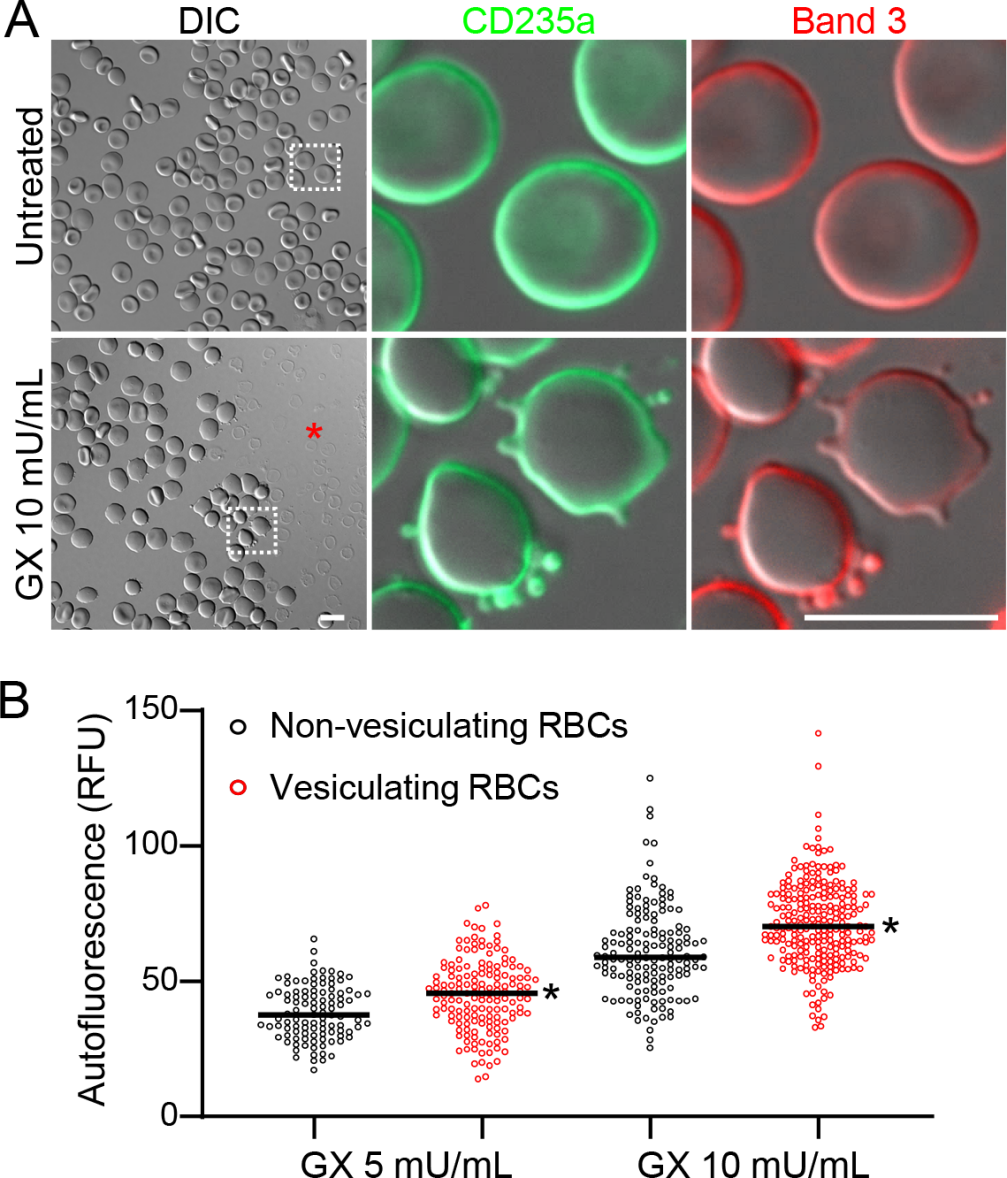
Formation of RBC microvesicles. **(A)** Representative differential interference contrast (DIC) and dual immunofluorescence imaging of Band 3 and CD235a in untreated and 10 mU/mL GX-treated RBCs after 24 hours. GX-treated RBCs exhibit membrane blebbing and shedding of CD235a- and Band 3-labeled microvesicles, typically adjacent to discrete areas containing accumulated lysed ghost cells (red asterisk). Dashed white boxes indicate regions of digital magnification. Scale bars = 10 µm. **(B)** Quantitative analysis of autofluorescence intensity comparing RBCs undergoing active vesiculation (red circles) with neighboring non-vesiculating RBCs (black circles) following treatment with 5 or 10 mU/mL GX for 24 hours. Relative fluorescence units (RFUs) are shown for individual RBCs (n = 100–200 cells per condition from 4 independent experiments). Black bars represent mean RFU values for each group. *p < 0.001 vs. non-vesiculating RBCs.

Microscopic examination of GX-treated RBC cultures revealed time-dependent vesiculation with release of vesicles staining for Band 3 and CD235a (**Figure 5A**). Microvesicle-forming cells were not uniformly distributed but instead clustered in discrete areas with vesiculating cells arranged in concentric formations around centrally located ghost cells. Semiquantitative image analysis of low magnification fields confirmed that such cluster formations were absent in control cultures but detectable at 12 hours and expanded in overall area by 24 hours across all GX concentrations (**Supplementary Figure 2**). Within these cluster regions, cells undergoing active vesiculation exhibited elevated mean autofluorescence compared to neighboring non-vesiculating cells. Mean RFU values ± SEM were 44.9 ± 1.0 versus 38.5 ± 0.97 for the 5 mU/mL group and 71.4 ± 1.0 versus 61.2 ± 1.4 for the 10 mU/mL group (p < 0.0001) (**Figure 5B**). These findings demonstrate that sustained low-level oxidant exposure drives progressive RBC vesiculation, while the spatial clustering pattern of vesiculating RBCs suggests that paracrine effects from lysed ghost cells may also contribute to this process.

### Vesiculation without phosphatidylserine externalization

**Figure 6:**
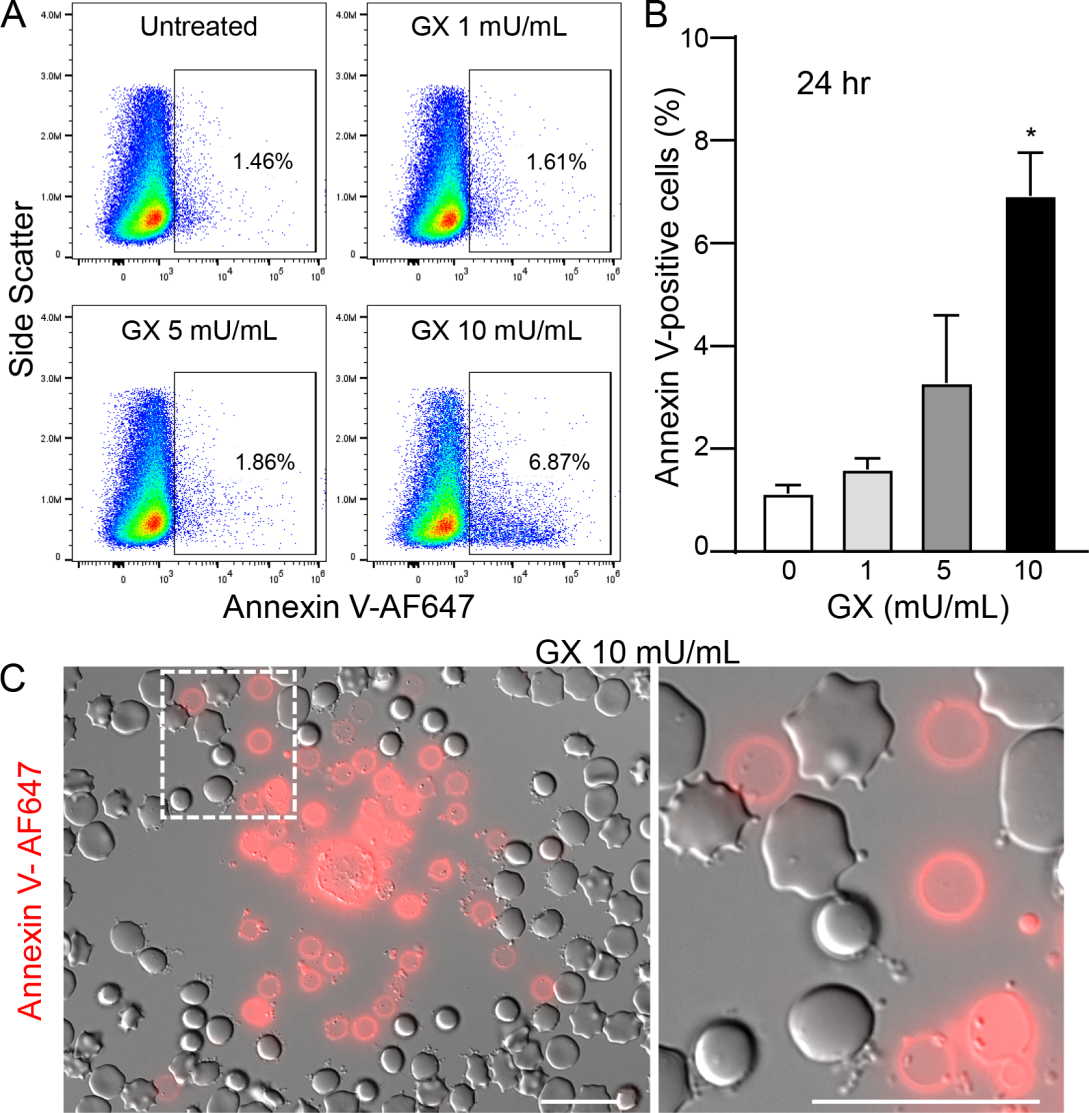
Phosphatidylserine externalization. **(A)** Flow cytometry analysis of Annexin V-AF647-positive cells following incubation with DPBS alone or in the presence of 1, 5, or 10 mU/mL GX for 24 hours. Dot plots show side scatter (SSC) versus Annexin V-AF647 fluorescence intensity. Representative data from three independent experiments are shown. **(B)** Quantitative analysis of % Annexin V-positive cells at 24 hours. Each value represents the mean ± SEM for a minimum of three independent experiments (n = 3 individual donors). *p < 0.05 vs. DPBS alone. **(C)** Representative fluorescence imaging of Annexin V-AF647 labeling after treatment with 10 mU/mL GX for 24 hours. GX-treated RBCs with intact membrane morphology or actively undergoing vesiculation show no PS externalization, nor do nearby shed microvesicles, whereas ghost cells are uniformly PS positive. Dashed white box denotes area of digital magnification. Scale bars = 20 µm.

PS externalization was analyzed by Annexin V binding using flow cytometry and fluorescence microscopy. At 24 hours post-treatment, flow cytometry revealed a modest concentration-dependent increase in Annexin V-positive cells that only reached statistical significance in the 10 mU/mL group (**Figures 6A, B**). Examination by fluorescence microscopy demonstrated that actively vesiculating RBCs as well as microvesicles budding or neighboring these cells exhibited no Annexin V binding (**Figure 6C**). In contrast, ghost cells and their associated vesicles showed robust Annexin V binding, likely reflecting PS exposure due to compromised membrane integrity (**Figure 6C**). Positive control treatment with ionomycin induced robust Annexin V binding on membrane-intact RBCs, validating the staining procedure for detecting surface-exposed PS (**Supplementary Figure 3**). These results suggest that the modest concentration-dependent increase in Annexin V-positive cells detected by flow cytometry in GX-treated samples reflects ghost cell accumulation rather than PS externalization on intact or actively vesiculating RBCs. Consistent with this interpretation, Annexin V-positive cells displayed SSC vs. FSC scatter characteristics suggestive of ghost cells, distinct from those of intact RBCs (**Supplementary Figure 4**). Taken together, these data support the idea that PS externalization does not precede vesiculation, and that increased Annexin V positivity only occurs after membrane failure in this model.

### Reduced RBC deformability and hemoglobin release

**Figure 7:**
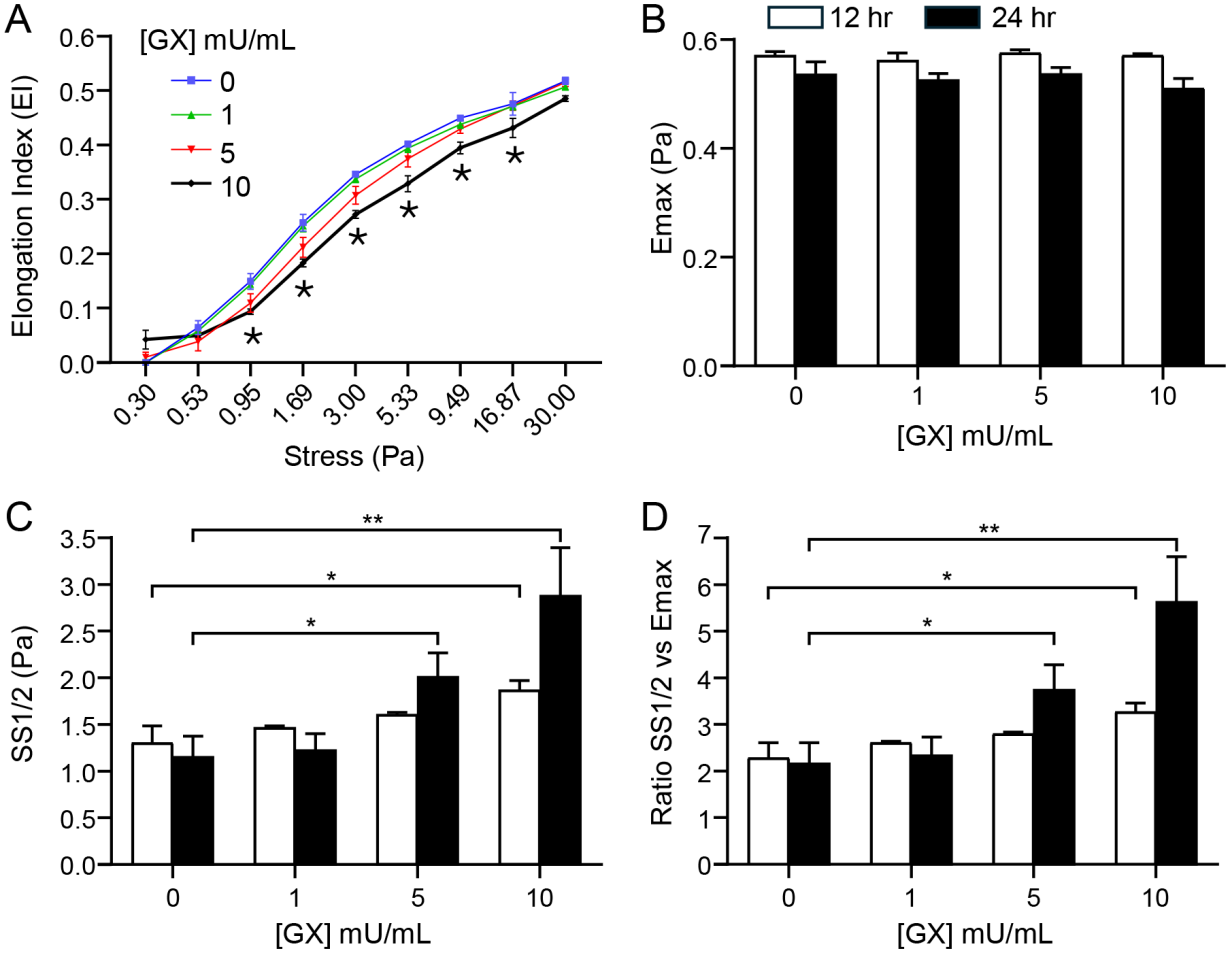
RBC deformability alterations following GX exposure. Untreated and GX-treated RBCs were analyzed by Lorrca ektacytometry. **(A)** Shear stress–elongation index (EI) curves obtained at 37 °C at nine shear stresses between 0.3 and 30 Pa after 24-hour treatment. Data are presented as the means ± SD, n = 4 individual donors for each group. **(B)** Maximum elongation (Emax), **(C)** shear stress at half-maximal elongation (SS1/2), and **(D)** the ratio of SS1/2 to Emax were calculated after 12 or 24 hours. *p < 0.05, **p < 0.001 vs. DPBS alone.

RBC mechanical properties were evaluated by ektacytometry after 12 and 24 hours of GX treatment. At 24 hours, GX-treated RBCs displayed concentration-dependent, downward-shifted elongation index (EI)–shear stress curves across 0.3–30 Pa compared with DPBS controls (**Figure 7A**), with the most pronounced impairment occurring in the 10 mU/mL group. Quantitative analysis revealed alterations in deformability parameters at both 12 and 24 hours. While maximum elongation (Emax) was not significantly affected by GX treatment (**Figure 7B**), shear stress at half-maximal elongation (SS1/2) increased significantly in a concentration-dependent manner (**Figure 7C**). The ratio of SS1/2 to Emax, an integrated measure of membrane rigidity, was also significantly elevated by GX in a concentration-dependent manner (**Figure 7D**). These results demonstrate that sustained extracellular H_2_O_2_ exposure compromises RBC mechanical flexibility.

**Figure 8:**
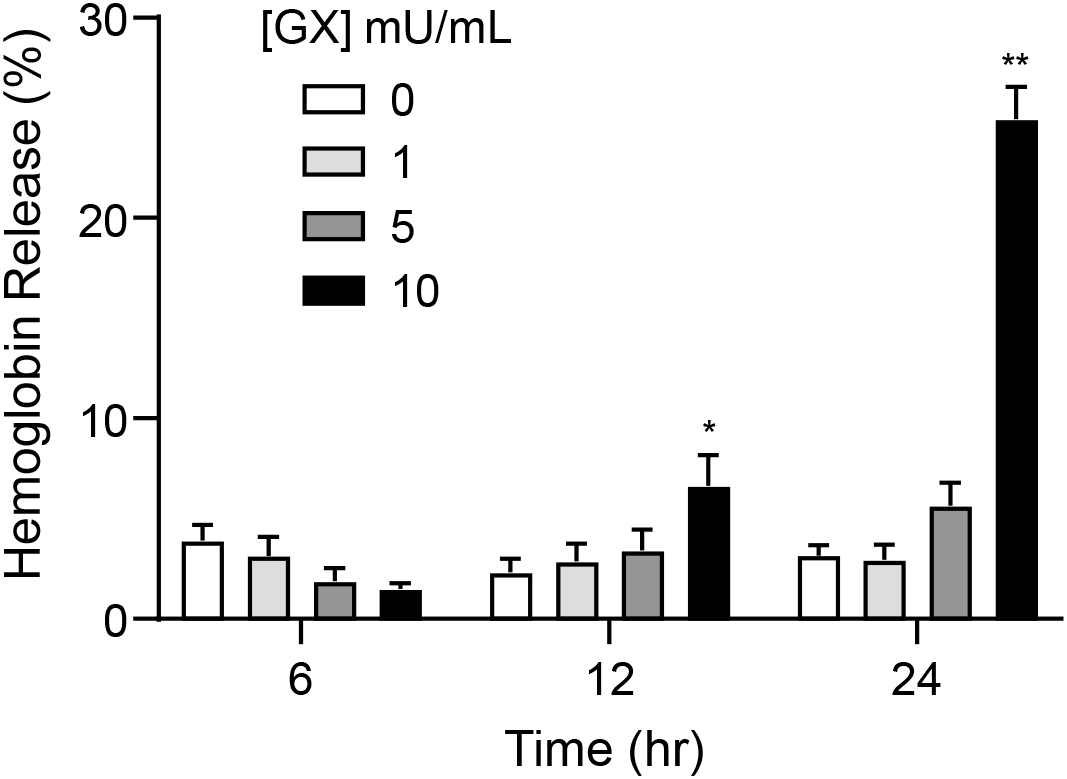
Hemoglobin release following GX exposure. Human RBCs were incubated with DPBS alone or in the presence of 1, 5, or 10 mU/mL GX. Relative hemoglobin release at each indicated time point is reported as the mean ± SEM for six independent experiments performed in duplicate or triplicate (n = 6 individual donors). *p < 0.05, **p < 0.0001 vs. DPBS alone.

Consistent with progressive membrane damage and reduced deformability, relative hemoglobin release increased in a GX concentration- and time-dependent manner (**Figure 8**). At 6 hours, hemoglobin release remained low across all groups (<4%). By 24 hours, 10 mU/mL GX induced approximately 25% hemoglobin release compared with 2–3% in DPBS controls (p < 0.0001). The 5 mU/mL group showed modest hemoglobin release (approximately 4–6% at 24 hours) that was not statistically significant (p = 0.36). The 1 mU/mL GX treatment showed no increase above control levels. Collectively, these findings demonstrate that GX-induced oxidative stress impairs RBC deformability and membrane integrity in a concentration-dependent manner, with concentrations ≥5 mU/mL producing measurable changes in deformability and 10 mU/mL causing substantial hemoglobin release indicative of membrane lysis by 24 hours.

## Discussion

This study employed an *in vitro* model with GX to generate sustained, low micromolar concentrations of H_2_O_2_, thereby mimicking oxidative stress conditions relevant to both physiology and pathophysiology. The primary objective was to characterize the progressive sequence of structural and functional alterations in human RBCs under this controlled oxidative challenge. Our findings establish a temporal hierarchy of oxidative injury: (1) rapid GSH depletion within 6 hours, (2) progressive methemoglobin accumulation, (3) increased oxidative autofluorescence with reduced cellular viability and/or membrane integrity, (4) impaired deformability, (5) vesiculation in susceptible cells, and (6) concentration-dependent hemoglobin release indicative of membrane lysis by ≥ 12 hours. Notably, PS externalization was not detected on intact or vesiculating RBCs at any timepoint, with Annexin V binding observed only on ghost cells after membrane disruption.

A key strength of this GX-based model is its ability to generate physiologically relevant H_2_O_2_ levels, which have been reported to be in the 0.8–6 µM range (Forman et al., 2016; Gaikwad et al., 2021). This approach stands in contrast to many previous studies that have relied on supraphysiological bolus additions of oxidants with or without catalase inactivation (Benincasa et al., 2025; Sinha et al., 2015; Russo et al., 2024; Snyder et al., 1985; Hale et al., 2011; Nagababu et al., 2003), and better reflects the progressive oxidative injury characteristics of the blood storage lesion (Rinalducci et al., 2012; Brun et al., 2021; D'Alessandro et al., 2019; Larkin et al., 2024). H_2_O_2_ levels in the 1 and 5 mU/mL groups remained stable throughout the 24-hour period, whereas the decline observed in the 10 mU/mL group after 8 hours coincided with the onset of hemoglobin release, suggesting that membrane lysis and subsequent release of intracellular catalase, peroxiredoxins, and other H_2_O_2_-degrading constituents into the culture medium accelerated extracellular H_2_O_2_ breakdown. This interpretation is further supported by the more stable H_2_O_2_ equilibrium observed in the 1 and 5 mU/mL groups, where minimal membrane lysis likely limited the release of H_2_O_2_-metabolizing enzymes. Additionally, glucose concentrations in culture supernatants remained above 4.5 mM after 24-hour incubation under all conditions tested (**Supplementary Figure 1B**), well above the Km for Glut-1-mediated glucose transport (~1–2 mM) and hexokinase-mediated glucose utilization (~0.1 mM) (Carruthers, 1990; Stocchi et al., 1982). This indicates that glucose availability was sufficient to sustain GX-mediated H_2_O_2_ generation throughout the experimental period, and further suggests that metabolic stress secondary to glucose depletion is not a confounding factor in this model.

The results illustrate a clear temporal and hierarchical progression of damage. One of the earliest events observed was the profound depletion of intracellular GSH, which, as a key non-enzymatic antioxidant in RBCs, renders the cell highly vulnerable to subsequent oxidative insults (Daraghmeh and Karaman, 2024; Orrico et al., 2023; Spinelli et al., 2025; Johnson et al., 1994; Winterbourn, 2026). At the low micromolar H_2_O_2_ concentrations generated in this model, peroxiredoxin-2 (Prx-2) — the most abundant thiol-containing protein in RBCs, present at an intracellular concentration of approximately 240 µM — serves as the primary enzymatic defense, operating efficiently at nanomolar to low micromolar H_2_O_2_ levels and relying on thioredoxin and NADPH for regeneration (Low et al., 2007; Orrico et al., 2023; Winterbourn, 2026). However, when Prx-2 becomes overwhelmed or hyperoxidized under sustained peroxide load, excess H_2_O_2_ spills over to the GSH system, placing an increased burden on glutathione peroxidase-1 (GPx-1) and accelerating GSH consumption. GPx-1 acts in concert with GSH to reduce H_2_O_2_, but its activity is directly dependent on GSH availability; moreover, both the glutathione system and the thioredoxin-dependent Prx-2 regeneration cycle compete for the same limited NADPH pool, such that Prx-2 failure further strains GSH regeneration by glutathione reductase. Consequently, the rapid GSH depletion observed here would progressively impair GPx-1 function, leaving a compromised Prx-2 and catalase as the remaining active defenses. Catalase, while highly efficient at elevated H_2_O_2_ concentrations, has a relatively high Km for H_2_O_2_ and is therefore less effective at the low micromolar levels sustained in this model, which may explain why GSH depletion occurs rapidly even at the lowest GX concentration tested (1 mU/mL). Together, this antioxidant hierarchy may account for the observed sequence in which GSH exhaustion precedes the progressive concentration-dependent increase in methemoglobin as downstream defenses are overwhelmed.

At the single-cell level, we established a strong inverse correlation between oxidative damage (RBC autofluorescence) and viability and/or membrane integrity (calcein retention). Although reduced calcein fluorescence reflects either esterase inhibition or membrane permeabilization, the assay nonetheless captures a biologically meaningful relationship between oxidative burden and cellular dysfunction. Future studies employing complementary membrane integrity assays will be needed to further delineate the relative contributions of these mechanisms. This oxidative injury manifests functionally as a significant impairment in RBC deformability, a critical adverse event that can impede microcirculatory transit (Larkin et al., 2024; Obeagu et al., 2024; Mohanty et al., 2014). Given that ektacytometry provides an ensemble average, the deformability data at the highest GX concentration could in principle be influenced by the presence of ghost cells and membrane debris; however, the low percentage of ghost cells identified by Annexin V flow cytometry suggests this had minimal impact on the overall interpretation of these results. Notably, at the intermediate exposure of 5 mU/mL GX, we observed extensive GSH exhaustion, methemoglobin formation and significant loss of deformability with minimal hemoglobin release, indicating that the observed mechanical impairment occurred in a predominantly intact RBC population. This underscores a distinct, vulnerable pre-lytic state wherein RBCs are biochemically and biomechanically compromised prior to complete membrane lysis. Such a window of vulnerability would be difficult to capture using acute, high-concentration bolus challenges.

Vesiculation has been proposed to serve dual biological functions: a damage response mechanism and a potential quality control process (Leal et al., 2018; Nguyen et al., 2016; Sudnitsyna et al., 2020). By selectively removing oxidatively damaged membrane components and proteins, vesiculation may temporarily preserve parent RBC function by maintaining a more favorable surface area-to-volume ratio and eliminating aggregated or modified proteins that could trigger premature clearance. However, excessive vesiculation compromises the overall surface area-to-volume ratio and contributes to the progressive loss of deformability. The presence of Band 3 in shed microvesicles with Band 3 clustering and oxidative modification serve as recognition signals for macrophage-mediated clearance *in vivo* (Badior and Casey, 2018), suggesting that microvesicle formation may represent an intermediate stage in the progression toward premature RBC removal from circulation. Our imaging data revealed morphological remodeling, including membrane blebbing and the formation of Band 3- and CD235a-positive microvesicles. Notably, microvesicle-forming RBCs were not uniformly distributed throughout the culture but rather appeared in discrete clusters, often in proximity to ghost cells, suggesting that factors released from lysed cells may create localized microenvironments conducive to membrane remodeling. Within these cluster regions, cells undergoing active vesiculation exhibited significantly elevated mean autofluorescence compared to neighboring non-vesiculating cells, indicating a higher oxidative burden in vesiculating RBCs and suggesting that vesiculation represents a threshold phenomenon requiring both oxidative damage and cell-specific susceptibility factors.

Contrary to expectations, PS externalization was not detected on intact RBCs undergoing oxidative stress, including those actively forming microvesicles, or on their shed microvesicles. The modest concentration-dependent increase in Annexin V-positive populations detected by flow cytometry likely reflects ghost cell accumulation rather than PS externalization on intact RBCs (**Figure 6**; **Supplementary Figure 4**). This demonstrates that under physiologically relevant oxidant exposure, membrane asymmetry is maintained until membrane lysis, indicating that PS externalization is not an early biomarker of oxidative damage in this model.

This uncoupling of vesiculation and PS externalization demonstrates that these processes represent distinct pathways of membrane remodeling that can be temporally and mechanistically separated. This finding challenges models that propose direct linkage between these processes and suggests that the specific nature and intensity of oxidative stress determine which membrane alterations predominate (Lang et al., 2012; Qadri et al., 2017; Sudnitsyna et al., 2020). Vesiculation may occur through localized cytoskeletal weakening and membrane budding driven by oxidative modification of spectrin-actin interactions, whereas PS externalization may require more extensive disruption of calcium homeostasis and energy metabolism. Testing these hypotheses will require direct measurement of cytoskeletal protein oxidation, calcium flux, ATP levels, and aminophospholipid translocase activity in vesiculating versus non-vesiculating cells under sustained oxidative conditions.

While this *in vitro* model provides valuable insights, several limitations should be acknowledged. The study was conducted under static conditions, which do not replicate the continuous shear stress and dynamic environment experienced by RBCs within the circulatory system. Our open culture system also differs from the closed, gas-permeable blood bag environment, where oxygen gradients, pH shifts, and metabolite accumulation create conditions distinct from our experimental setup. The experimental buffer system also lacks the full complement of plasma proteins, metabolites, or storage solution components that could modulate RBC antioxidant capacity and injury response. The model also focuses exclusively on H_2_O_2_-mediated stress, whereas the *in vivo* environment involves a complex mixture of reactive species. Additionally, the experiments were performed using RBCs from healthy volunteers, and the response to oxidative stress may differ significantly in RBCs from individuals with hematological disorders or in cells from aged, stored blood units. Another consideration is the experimental cell density of 1.65–1.75 × 10^6^ cells/mL (~0.05% hematocrit), which is substantially lower than physiological (~40–45%) or blood bank storage (~60-70%) conditions. This reduced cell density was adopted to enable single-cell imaging analyses that are not feasible at physiological hematocrits; accordingly, GX concentrations were carefully titrated at this density to generate physiologically relevant, low-micromolar H_2_O_2_ levels. Given that the oxidant:antioxidant ratio is a function of cell number, the absolute kinetics and thresholds of oxidative injury reported here may differ from those at physiological or blood bank storage hematocrits. Finally, the 24-hour timeframe captures acute oxidative responses but does not recapitulate the cumulative damage that accrues over weeks of blood bank storage.

Despite these limitations, the findings suggest several avenues for future research. This GX-based model serves as a robust platform for investigating the molecular mechanisms underlying the blood storage lesion and for screening novel antioxidant compounds designed to improve the quality of transfused blood. Future studies should incorporate physiological shear stress using microfluidic systems to better recapitulate *in vivo* conditions and determine whether mechanical forces modulate the threshold for microvesicle formation or PS externalization (Kim et al., 2022). Multi-omics approaches, including proteomics and lipidomics, could provide comprehensive molecular profiles of RBCs at different stages of oxidative damage, potentially identifying novel biomarkers and therapeutic targets (D'Alessandro, 2023). Finally, hyperspectral darkfield microscopy may also offer a powerful approach for detecting non-lytic membrane changes in oxidatively stressed RBCs at the single-cell level (Vartian et al., 2026).

In conclusion, this study establishes a physiologically relevant model of sustained low-level oxidative stress and defines a temporal hierarchy of RBC damage that progresses from antioxidant depletion through functional impairment to structural failure. The distinct patterns of vesiculation and PS externalization observed under sustained versus acute oxidative conditions highlight the importance of using physiologically relevant experimental models for understanding RBC biology and developing clinically meaningful quality assessment tools.

## Data Availability

The original contributions presented in the study are included in the article/**Supplementary Material**, further inquiries can be directed to the corresponding author.

## Supplementary material

Supplementary Figure 1 **(A)** GX-mediated H₂O₂ accumulation in the presence or absence of RBCs. Culture dishes containing DPBS were treated with 10 mU/mL GX with or without RBCs as described in the Materials and Methods. H₂O₂ concentrations were measured at the indicated time points over 24 hours. Each value represents the mean ± SEM for 5 independent experiments performed in triplicate. **(B)** Glucose concentrations in RBC cultures. Extracellular glucose concentrations were measured in culture supernatants collected after 24-hour incubation of RBCs in DPBS alone or in the presence of 1, 5, or 10 mU/mL GX, using the Amplex Red Glucose/Glucose Oxidase Assay Kit (Cat. No. A22189, Thermo Fisher Scientific) according to manufacturer’s instructions. Supernatants were diluted 1:200 prior to assay, and absorbance at 560 nm at the 30-minute time point was used for quantification against a glucose standard curve. Each value represents the mean ± SEM for 7 independent experiments performed in duplicate (n = 7 individual donors). *p < 0.05, **p < 0.0001 vs. DPBS alone.

Supplementary Figure 2 Semiquantitative analysis of microvesicle-forming cell cluster area in GX-treated RBC cultures. **(A)** Representative low magnification fields (10x objective) showing an untreated control monolayer with no visible cluster formations and a 10 mU/mL GX-treated culture at 24 hours exhibiting characteristic concentric cluster formations (red asterisks) surrounding centrally located ghost cells. **(B)** Semiquantitative analysis of percent cluster area in untreated and GX-treated RBC cultures (1, 5, and 10 mU/mL GX) at 12 and 24 hours. For each coverslip, a minimum of 15 low power field images were captured. Cluster areas within each image were delineated and measured using the Region of Interest (ROI) Manager in ImageJ; the sum of all cluster areas was then divided by the total image area to yield a % cluster area value per image. These values were averaged across all 15 images to derive a mean % cluster area per coverslip. The per-coverslip means were then averaged across 4 independent experiments, each performed in duplicate or triplicate (n = 4 individual donors), and are plotted as the group-level mean % cluster area ± SEM. Statistical comparisons were made using a one-way ANOVA with Dunnett's post-hoc test, comparing each GX-treated group against the untreated control. *p < 0.01, **p < 0.001, ***p < 0.0001 vs. DPBS alone.

Supplementary Figure 3 Ionomycin induces PS externalization in human RBCs. Representative fluorescence imaging of Annexin V-AF647 labeling in untreated RBCs or after treatment with 1 µM ionomycin for 90 minutes. Annexin V binds to PS exposed on the outer membrane leaflet, indicating calcium-induced membrane alterations. Dashed white box denotes area of digital magnification. Scale bars = 10 µm.

Supplementary Figure 4 **(A)** Annexin V-AF647 fluorescence density scatter plots of untreated and GX-treated RBCs following 24 hours of incubation. Gate P1 denotes the total RBC population based on characteristic FSC and SSC parameters, while gate P2 denotes the Annexin V-positive subpopulation within P1. The percentage of events within P1 (% P1) is indicated for each condition. **(B)** Corresponding SSC versus FSC density scatter plots of the total RBC population (P1) for each condition. **(C)** Corresponding SSC versus FSC density scatter plots of the Annexin V-positive subpopulation (P2) for each condition. Annexin V-positive cells from the 10 mU/mL GX-treated sample accumulate in a subpopulation with lower FSC and SSC values, consistent with ghost cells that have lost hemoglobin content and internal complexity, compared to intact RBCs in the P1 population.
