# Supplementary figures and images for: Sustained low micromolar hydrogen peroxide exposure induces sequential red blood cell dysfunction
Source: Front Physiol. 2026 Jul 10;17:1860905. doi: 10.3389/fphys.2026.1860905 (PMC13397108; doi:10.3389/fphys.2026.1860905)

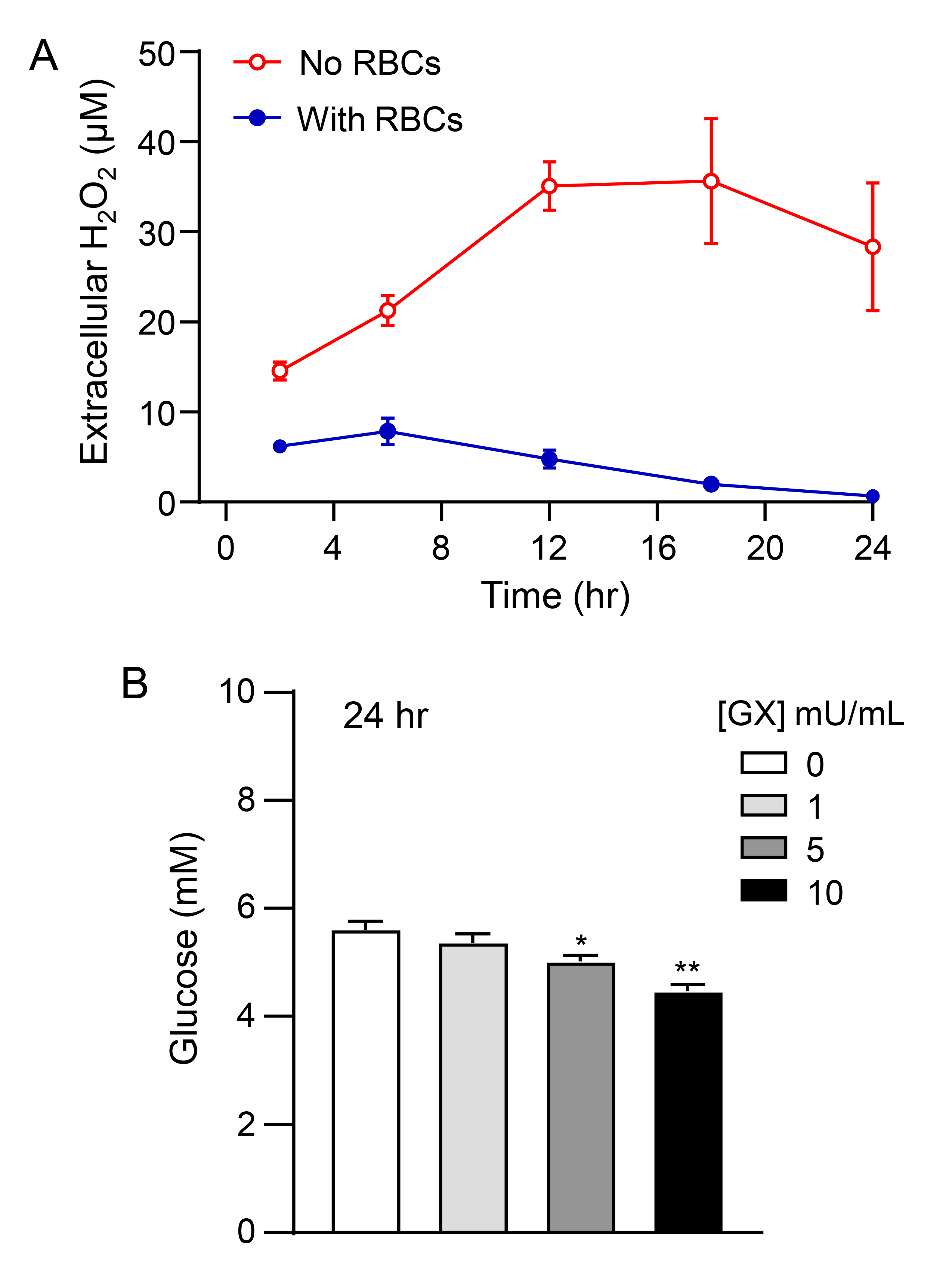

Supplement: Supplementary Figure 1 — (A) GX-mediated H₂O₂ accumulation in the presence or absence of RBCs. Culture dishes containing DPBS were treated with 10 mU/mL GX with or without RBCs as described in the Materials and Methods. H₂O₂ concentrations were measured at the indicated time points over 24 hours. Each value represents the mean ± SEM for 5 independent experiments performed in triplicate. (B) Glucose concentrations in RBC cultures. Extracellular glucose concentrations were measured in culture supernatants collected after 24-hour incubation of RBCs in DPBS alone or in the presence of 1, 5, or 10 mU/mL GX, using the Amplex Red Glucose/Glucose Oxidase Assay Kit (Cat. No. A22189, Thermo Fisher Scientific) according to manufacturer’s instructions. Supernatants were diluted 1:200 prior to assay, and absorbance at 560 nm at the 30-minute time point was used for quantification against a glucose standard curve. Each value represents the mean ± SEM for 7 independent experiments performed in duplicate (n = 7 individual donors). *p < 0.05, **p < 0.0001 vs. DPBS alone. [file Image1.tif]

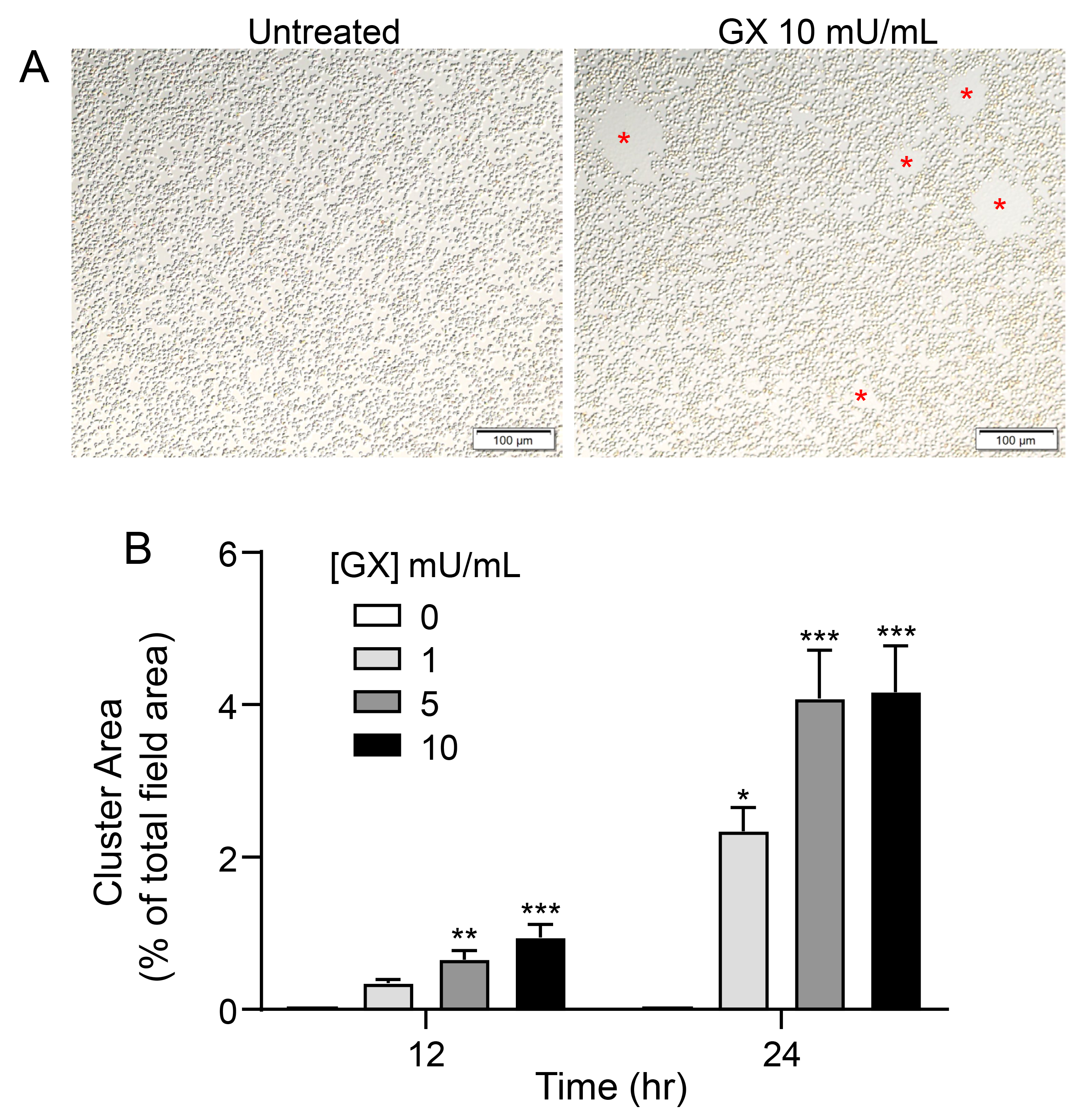

Supplement: Supplementary Figure 2 — Semiquantitative analysis of microvesicle-forming cell cluster area in GX-treated RBC cultures. (A) Representative low magnification fields (10x objective) showing an untreated control monolayer with no visible cluster formations and a 10 mU/mL GX-treated culture at 24 hours exhibiting characteristic concentric cluster formations (red asterisks) surrounding centrally located ghost cells. (B) Semiquantitative analysis of percent cluster area in untreated and GX-treated RBC cultures (1, 5, and 10 mU/mL GX) at 12 and 24 hours. For each coverslip, a minimum of 15 low power field images were captured. Cluster areas within each image were delineated and measured using the Region of Interest (ROI) Manager in ImageJ; the sum of all cluster areas was then divided by the total image area to yield a % cluster area value per image. These values were averaged across all 15 images to derive a mean % cluster area per coverslip. The per-coverslip means were then averaged across 4 independent experiments, each performed in duplicate or triplicate (n = 4 individual donors), and are plotted as the group-level mean % cluster area ± SEM. Statistical comparisons were made using a one-way ANOVA with Dunnett's post-hoc test, comparing each GX-treated group against the untreated control. *p < 0.01, **p < 0.001, ***p < 0.0001 vs. DPBS alone. [file Image2.tif]

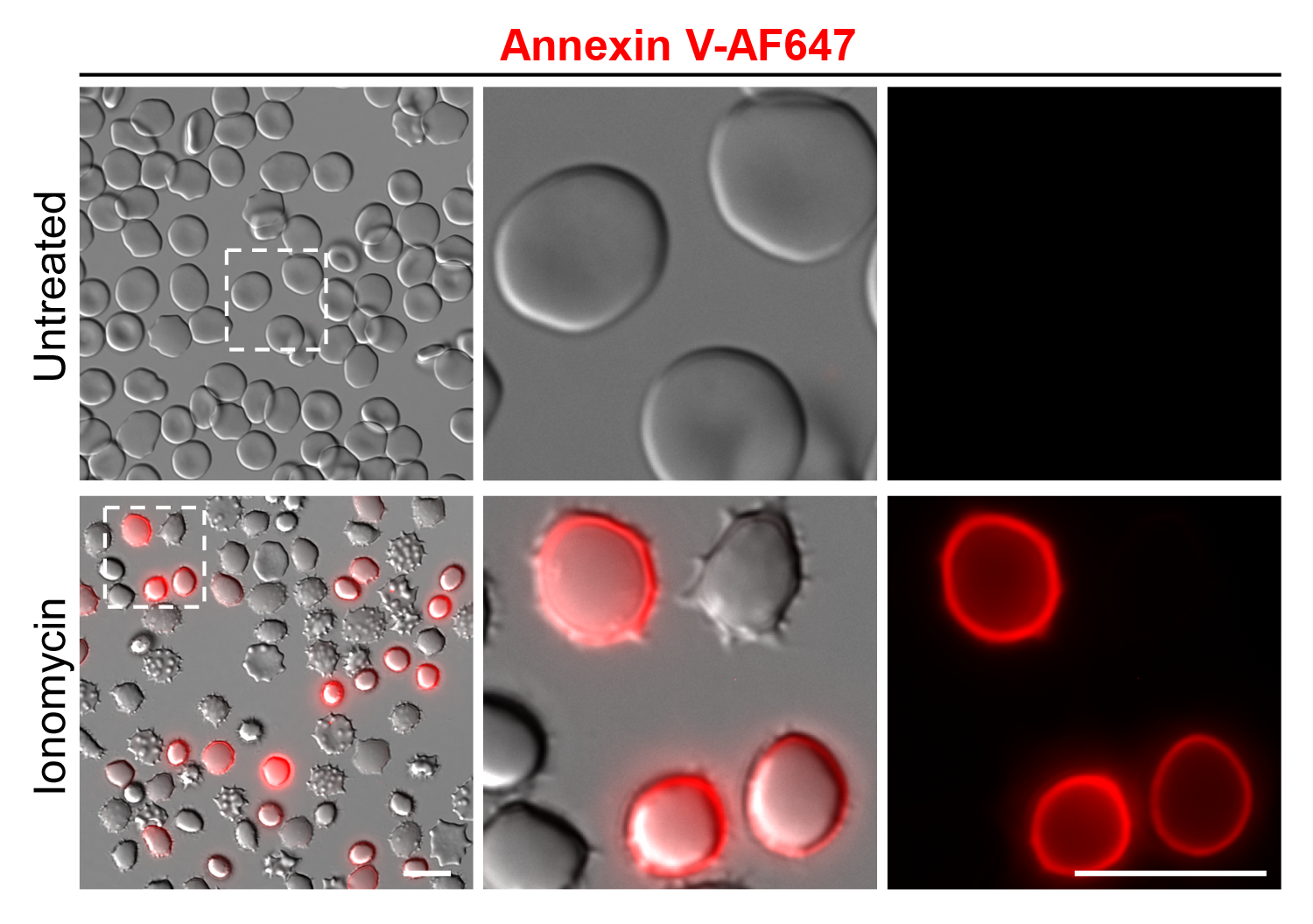

Supplement: Supplementary Figure 3 — Ionomycin induces PS externalization in human RBCs. Representative fluorescence imaging of Annexin V-AF647 labeling in untreated RBCs or after treatment with 1 µM ionomycin for 90 minutes. Annexin V binds to PS exposed on the outer membrane leaflet, indicating calcium-induced membrane alterations. Dashed white box denotes area of digital magnification. Scale bars = 10 µm. [file Image3.tif]

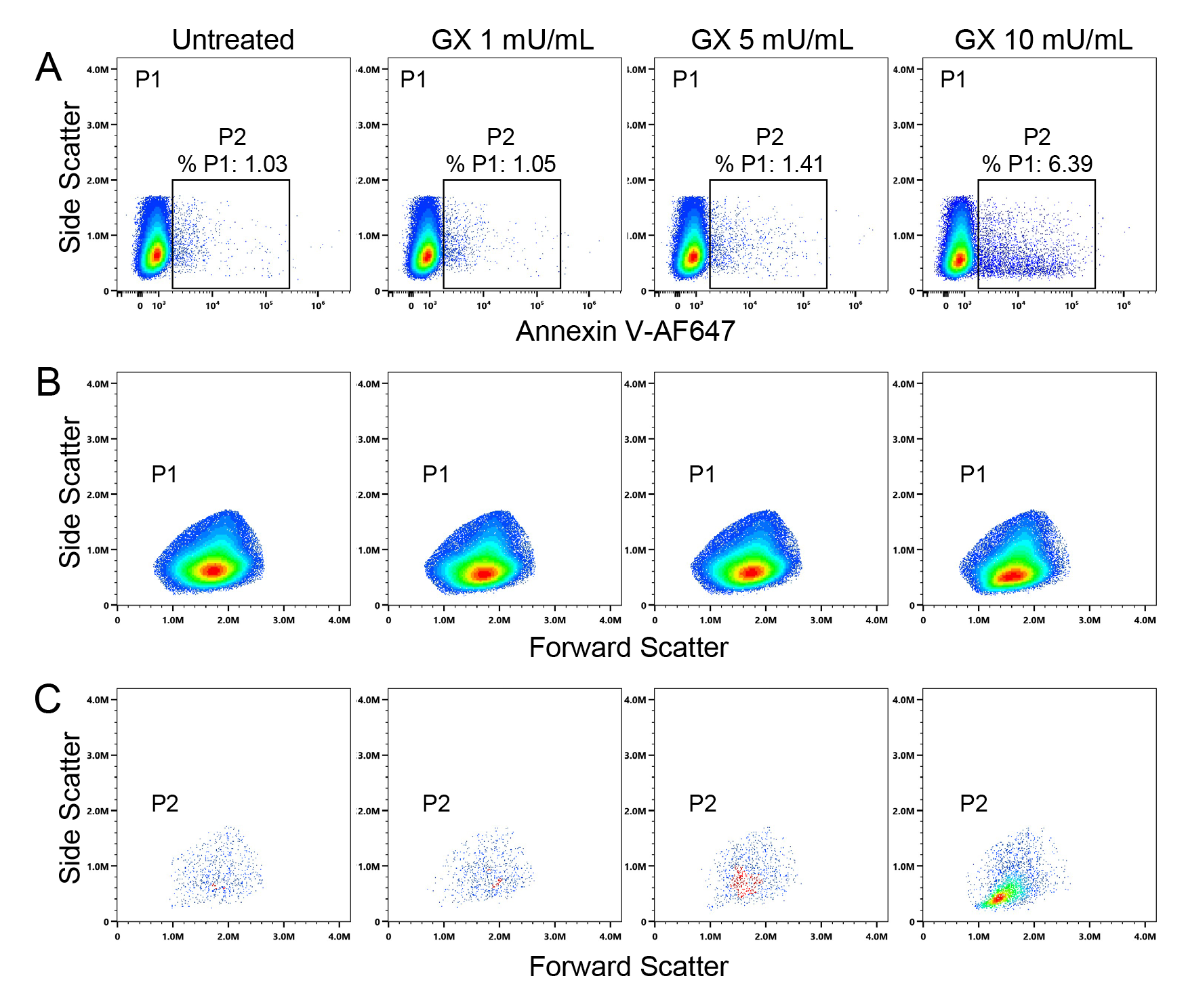

Supplement: Supplementary Figure 4 — (A) Annexin V-AF647 fluorescence density scatter plots of untreated and GX-treated RBCs following 24 hours of incubation. Gate P1 denotes the total RBC population based on characteristic FSC and SSC parameters, while gate P2 denotes the Annexin V-positive subpopulation within P1. The percentage of events within P1 (% P1) is indicated for each condition. (B) Corresponding SSC versus FSC density scatter plots of the total RBC population (P1) for each condition. (C) Corresponding SSC versus FSC density scatter plots of the Annexin V-positive subpopulation (P2) for each condition. Annexin V-positive cells from the 10 mU/mL GX-treated sample accumulate in a subpopulation with lower FSC and SSC values, consistent with ghost cells that have lost hemoglobin content and internal complexity, compared to intact RBCs in the P1 population. [file Image4.tif]
